# Orocervical foetus-in-foetu with prenatal sonographic diagnosis: a case report

**DOI:** 10.1186/1752-1947-2-362

**Published:** 2008-12-04

**Authors:** Kolawole T Braimoh, Adekunle Y Abdulkadir, Rabiu O Balogun

**Affiliations:** 1 Department of Radiology, University of Ilorin Teaching Hospital, P.M.B. 1459, Ilorin, Kwara State, Nigeria; 2 Department of Obstetrics and Gynaecology, University of Ilorin Teaching Hospital, P.M.B. 1459, Ilorin, Kwara State, Nigeria

## Abstract

**Introduction:**

Foetus-in-foetu is a very rare congenital abnormality where a malformed foetus is included within the body of another foetus. Less than 200 cases have been reported with over 80% occurring in the abdomen. Only three cases of cervical foetus in foetu have been reported. The present case of giant orocervical foetus-in-foetu appears to be an index case.

**Case presentation:**

This is a report of an extremely rare orocervical foetus-in-foetu with grotesque oddity diagnosed on prenatal ultrasonography at 35 weeks gestational age in a 28-year-old, G2P1+0, Nigerian woman who was unsure of her last menstrual date or month. The included foetus had two eyes, cranium, nose, long bones and a spine. The mother's attempts at vaginal delivery rather than the elective Caesarean delivery she was offered resulted in obstructed labour and intrauterine foetal demise.

**Conclusion:**

Giant cervical foetus-in-foetu is extremely rare. It could result in obstructed labour if vaginal delivery is attempted.

## Introduction

Foetus-in-foetu (FIF) is a rare abnormality where a malformed foetus is found within the body of a normally developing foetus [1-3]. Less than 200 cases have been reported in the literature to date [1,2]. Over 90% of the reported cases emanated from Asia, Europe and North America. The majority of the reported cases are intra-abdominal in location [1,2]. We found only three cases of cervical FIF documented in the electronic literature. Thus, the present case of giant orocervical FIF appears to be an index case and its extreme rarity renders it an important addition to the disease entity.

## Case presentation

A 28-year-old Nigerian woman, who was gravida 2, para 1, and who was not sure of her last menstrual date or last month of menstruation, was referred for obstetrics ultrasound scan for dating and foetal well-being. She had no medical history of note and was not on any specific drugs during this pregnancy. She had a healthy 2-year-old boy. She had come to the booking clinic with an advanced pregnancy estimated to be 39 to 40 weeks gestation by the obstetricians. She was in a good state of health (BP = 110/70 mmHg, respiratory rate 18 cpm and pulse rate 80 bpm).

**Figure 1:**
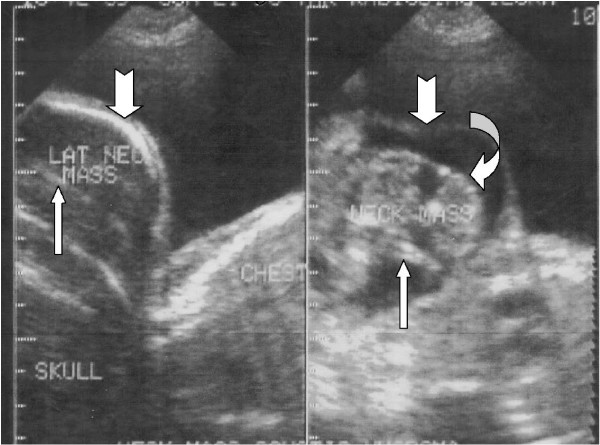
**Sonograms of the foetal neck mass, midline longitudinal scan (left image) and left paramedian scan (right image), showing a large cyst that resembles a gestational sac (notched arrow).** Note the central irregular solid tissue containing long bone (white arrow) and vertebral bone (curved arrow).

Transabdominal ultrasound examination showed a singleton live intrauterine foetus in longitudinal lie and presenting cephalic. The amniotic fluid index was elevated (55 mm 52 mm, 62 mm, 68 mm). The foetus had a complex cystic lower jaw/neck mass (Figure 1). The mass was well encapsulated and contained some echogenic long bones. The differential diagnosis included lymphangioma, teratoma, and a branchial cleft cyst. Measurement of biparietal diameter was made difficult by this mass. Hence, foetal age was estimated from femur length and abdominal circumference and both corresponded to 36 weeks gestation. She was planned for elective Caesarean delivery, but she defaulted for vaginal delivery at home only to represent when labour had been obstructed for 72 hours and foetal demise had occurred.

She was brought in exhausted, dehydrated, tachycardic (102 bpm) and tachypnoeic (28 cpm). She was febrile (39°C) but not pale. Foetal heart sound was not heard. The foetal head was engaged and os was fully dilated. An impression of obstructed labour with intrauterine fetal death presumably due to the giant neck mass was made.

**Figure 2:**
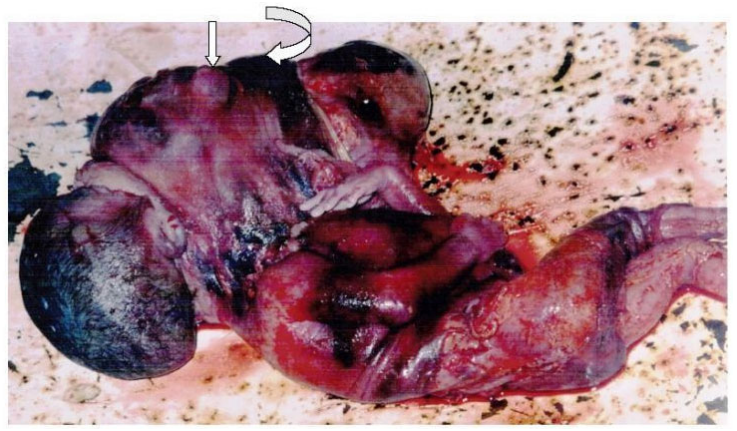
The baby with a giant orocervical foetus-in-foetu. Note the bulging eye (straight arrow) and a nasal ridge (curved arrow).

She had an emergency Caesarean section and a macerated foetus was delivered (Figure 2).

The baby weighed 3950 g and showed no dysmorphic features except for a large, mostly firm mass on the neck involving the chin (Figure 2). The mass contained eyes, nose and skull all surrounded by a membrane-like thin skin.

Pathologic examination of the mass was hindered by cultural taboo, which led to the patient and her family declining consent. This foetiform structure was determined to be a foetus in foetu because of the high degree of organogenesis, which included cranium, eyes, nose, long bones and the presence of a vertebral axis.

## Discussion

Foetus-in-foetu (FIF) occurs in about 1/500,000 deliveries with a male/female ratio of 2:1 [1,2].

The embryogenetic mechanism remains uncertain but it is generally believed that there is a continuum between FIF and teratomas [4,5]. Some investigators have hypothesized that FIF results from a modified process of twinning and have traced the progression from normal twins to conjoined symmetrical twins, through malformed external parasitic foetuses, foetal inclusion and finally to teratoma [4-6]. Supporting this theory were Spencer's [4] observations that FIF and teratomas are: increased in families with a history of twinning; both may coexist; common site of occurrence; and FIF may contain multiple foetuses. Spencer [4] proposed primary cardiac malformation in the aetiogenesis with secondary disruption in the development of the brain because rarely, if ever, was a functional heart or a competent brain found in any FIF.

However, a case of FIF with a pulsating single chamber heart has been documented, which negates the primary acardiac theory [3]. Again, in the opinion of Rahman *et al*. [1] who in 2007 reviewed 161 reported cases of FIF, Spencer's theories, though appearing encompassing, cannot explain why southwestern Nigeria with the highest rate of twinning in the world has no documented case of FIF. Whether this is a case of an extreme rarity in this region or non-reporting is not known.

Thus, Beaudoin *et al*.'s [7] theory of defective implantation during the second week of development leading to the invasion of a second embryo (that becomes a homunculus) into the extra-embryonic mesenchyma of the other foetus (the autosite) instead of the uterine wall favourably explains the various sites of FIF. The possibilities following defective implantation are: (1) primary gastrulation could occur normally in both leading to two primitive streaks; (2) the homunculus may fail to differentiate its own extra-embryonic mesenchyma into a cardiogenic zone leading to acardia; (3) the inductor's signal for the parasitic notochord may be disabled by those of the surrounding host leading to absent axial skeleton; and (4) some of the parasitic cells submitted to impaired induction may develop into teratomas or multiple foetiform structures [4,7]. These hypotheses can explain the orocervical location of the FIF in our patient and the associated organ differentiations that included skull, eyes, nose, long bones and the presence of a vertebral axis.

Nearly all body parts have been identified in FIF but it is the presence of a vertebral column at imaging or histopathological examination that secures the diagnosis [1-5]. The identified body parts (skull, eyes, long bones, nose and a spine) in our patient on gross examination and at ultrasonography, best define it as FIF rather than teratoma while the near total inclusion of the malformed foetus is rather of FIF than a cervical conjoined twin.

Very few cases of either cervical or oral FIF have been reported [8-11]. The oropharyngeal FIF reported by Kapoor [8] was the only case found to be similar to ours. Since Rahman *et al*.'s review of FIF in 2007, we have found only nine additions in the literature and we have summarized the various sites of occurrence of the total 170 reported cases to illustrate the rarity of this case (Table 1).

**Table 1:** Distribution of published cases of FIF by site of occurrence (1806 to July 2008)

Site of occurrence	Frequency (n)	Percentage (%)	Valid percentage (%)
Intra-abdominal	94	55.3	80.3
Intracranial	12	7.1	10.1
Chest	3	1.8	2.5
Neck	3	1.8	2.5
Mouth	3	1.8	2.5
Scrotal sac	3	1.8	2.5
Sacral/pelvis	1	0.6	0.6
Missing*	51	30	**100**
**Total**	**170**	**100**	**-**

*Missing is reported cases for which the abstract and the full paper could not retrieved or the abstract contains no information on the site of the FIF.

Prenatal diagnosis of FIF, which has been made possible by ultrasonography and magnetic resonance imaging (MRI), can allow for counselling of the parents and assists the obstetricians, neonatologists, and paediatric general surgeons in planning the perinatal and postnatal management [6,12]. Prenatal ultrasonographic evaluation of our patient identified an orocervical mass having a skull, eye, vertebral column and long bones (Figure 1). These and the phenotypic appearance of the macerated foetus (Figure 2) sum up our diagnosis of FIF.

Foetus-in-foetu is generally known to be benign and shows a good response to enucleation [1,2].

## Conclusion

Orocervical FIF is extremely rare. A giant FIF could result in obstructed labour if vaginal delivery is attempted.

## Consent

Written informed consent was obtained from the patient for publication of this case report and any accompanying images. A copy of the written consent is available for review by the Editor-in-Chief of this journal.

## Competing interests

The authors declare that they have no competing interests.

## Authors' contributions

KTB participated in acquisition of data, images and manuscript revision. AYA performed the literature search, drafting, writing and revision, and aquired relevant data relating to patient, reviewed the literature, carried out the analysis and interpretation of data that generated the final table to enrich the intellectual content of this case., ROB carried out image acquisition, design and revision. All authors read and approved the final manuscript.
